# Not just an information-delivery tool. An ethnographic study exploring Danish GPs’ perspectives on and experiences with the relational potential of email consultation

**DOI:** 10.1080/02813432.2020.1843939

**Published:** 2020-11-13

**Authors:** Elisabeth Assing Hvidt, Jens Søndergaard, Maja Klausen, Anette Grønning

**Affiliations:** a Research Unit of General Practice, Department of Public Health, University of Southern Denmark, Odense, Denmark; b Department for the Study of Culture, University of Southern Denmark, Odense, Denmark

**Keywords:** Email consultation, doctor–patient relationship, general practice, interactive health communication, Denmark

## Abstract

**Objective:**

To explore GPs’ perspectives on and daily experiences with the relational potential of email consultations.

**Design:**

Qualitative study with data from participant observation and semi-structured interviews

**Setting:**

General practice setting in Denmark

**Subjects:**

Practice personnel from four clinics were observed and 16 GPs (seven women and nine men, between 35 and 70 years of age) interviewed. Field notes and interview data were analysed using an inductive thematic analysis approach.

**Main outcome measures:**

Main themes and subthemes reporting GPs’ perspectives on and experiences with the relational potential of email consultations.

**Results:**

The analyses showed that due to perceived interpretational shortcomings, the GPs generally experienced email consultation as unsuitable for communication about relational, socio-emotional and sensitive matters. In doctor–patient relationships founded on mutual knowledge and trust, the email consultation was however used as a supportive communication channel, as a way for the patient to express emotions and affect and for the GP to proactively show interest and compassion towards the patient.

**Conclusion:**

Email consultations were highly context-variant. Within continuing relationships and in conjunction with face-to-face consultations, email consultation was used for supportive communication holding the potential for maintaining, strengthening and/or dissolving the GP-patient relationship. Therefore, email consultation is not simply an information-delivery tool but also holds more explicit relational potentials.

**KEY POINTS**
- Overall, the GPs perceived email consultation as unsuitable for non-medical, relationship-oriented purposes.
- Nonetheless, the GPs experienced that email consultations oftentimes comprised communication about relational and socio-emotional issues.
- Knowledge of the patient was a vital factor for the GP’s comfort in and acceptability of relational functions of email consultation.
- Email consultation is not simply an information-delivery tool as it holds the potential for maintaining, strengthening and/or dissolving the GP-patient relationship.

## Introduction

Email consultation was introduced in healthcare in the late nineties as a cost-effective and convenient means of obtaining quick access to healthcare and as a way to meet the challenge of an increasing healthcare demand [1]. Given the complex nature of the doctor–patient relationship, email consultations were never intended to mediate relational exchanges, such as socio-emotional or affective expressions (anger, concern, anxiety, reassurance, empathy or partnership) but for simple, task-oriented functions such as: prescription refills, communicating laboratory results and informational tasks [2]. However, studies investigating the content of email consultation have found that doctors and patients also frequently communicate about socio-emotional issues, expressing positive and negative affect [2–4]. This has made scholars speculate about the relational and socio-emotional potential of the medium within the context of the personal doctor–patient relationship [3]. Several media- and health-communication scholars argue that email consultation has the potential to support the doctor–patient relationship by providing a medium through which patients can express worries and concerns, and physicians can provide reassuring and empathic responses [5,6]. However, almost no research has been conducted that focuses on the type of health communication through email consultation that goes beyond the typical task-oriented communication and that serve non-medical, relationship-oriented purposes [3]. Our approaches for conceptualizing relational, socio-emotional and affective interaction in email consultations, and for engaging with and analyzing the data, are inspired by socio-technical perspectives on how technology, social practices and interactions are co-constructed and interdependent [7,8]. In general, these perspectives challenge traditional deterministic views of technologies as passive and decontextualized objects that serve purely instrumental or task-oriented tasks [9,10]. In this context, affect is conceptualized as the physiological powers that produce and give rise to emotions (e.g. anger, joy, anxiety) and that can be transmitted through technologies and influence interaction and relationships [11,12]. Taking a socio-technical approach to email consultation thus highlights the potential of email consultation as a medium for human expression and interaction.

Our study context is general practice in Denmark which has experienced an increase of more than 20% of patient encounters during the past 16 years [13]. Meanwhile, the number of GPs has decreased by approximately 6% since 2007 [14]. As part of a development towards a Danish general practice environment that meets demands to be cost-effective, email consultation was made an obligatory service in 2009 in order to ‘increase efficiency and quality through the digitisation of health care’ (Doctor’s Agreement, 2010). A decade later, the use of email consultation in Denmark has rapidly increased from 1.3 million consultations in 2008 to 7.2 million consultations per year in 2018, corresponding to 21% of all GP consultations whereas the telephone consultation volume has decreased from 14.3 million in 2009 to 9 million in 2019 [15,16]. The increase in email consultations might result from the mandatory nature of email consultation (the GP must answer the patient within a maximum of five working days) and from overall demand from patients who value convenient, round the clock, untriaged access to the GP [17]. Furthermore, Denmark is one of the countries in the world that for many years has been the first and fastest to invest in digitalization, and where citizens, businesses and the public sector have been seen to exploit the opportunities more than in other developed countries [18]. A study found that Denmark had the highest number of email consultations sent/received in Europe [19].

Against this backdrop, the aim of this study is to analyze GPs’ daily experiences with the relational potential of email consultations, focusing on socioemotional exchanges and affective expressions. This type of communication, although not exclusively, might play a role in developing, maintaining and/or dissolving the doctor–patient relationship [2].

## Design, material and methods

### Setting

General practice in Denmark serves as a first-contact access point to the fully tax-financed Danish healthcare system that offers almost all services free of charge to citizens, including email consultation. The GPs may refer patients to another specialist treatment. About 98% of all Danish citizens are listed with a GP (in most cases of their own choosing), and a GP usually has a patient list of around 1,600 patients [20]. The list system enables the GP to develop a better knowledge of the individual patient (continuity of care) and knowledge of the family. In 2018, there were 3,402 GPs in Denmark organised in 1,922 practices, where approximately one third was single-handed practices and two-thirds were shared practices [14]. GPs in Denmark work under a contract with the public funder in a mixed capitation and fee-for-service system, receiving reimbursement for every consultation [20].

### Email consultation in general practice in Denmark

Email consultation is accessed through the clinics’ website where patients are required to log into a separate secure web messaging system with their patient identification in order to send and receive messages. The emails that the patients can write are unstructured in their format, allowing the patient to enter open-ended free text. However, the messaging software system of the clinic sets a limit of a maximum of 500 characters. Most practice websites state a recommended use of email consultations for communication of test results, renewal of prescriptions and short messages yielding yes/no answers. Every message is encrypted and automatically integrated into the patient’s medical record and GPs receive a reimbursement of 43 Danish Kroners for each email consultation per patient per day, (equivalent of approximately 6 US Dollars).

### Data and sample

The data collection for this study was part of a larger qualitative research project exploring the potential of patient–physician email communication. In the present article, we build on data from participant observation conducted in four general practice clinics by the first author from January to June 2019 and on semi-structured, individual interviews with GPs also conducted by the first author from January to September 2019.

### Participant observation

The four clinics were selected based on their geographical location (urban and rural) and practice types (group or single-handed practices). The GPs in three of the four clinics were contacted by email and known by the first author on beforehand from earlier research. The GP from the fourth clinic was approached through a colleague working in the clinic. None of the GPs were pioneers in email consultations, nor did they possess any special knowledge about it.

The first author observed both doctor- and nurse-led consultations and spent time in lunchrooms and waiting rooms. Observations took place on seven different working days. The aim of the observations was to gain insight into all consultation forms (face-to-face, telephone and email consultation) and into how email consultations were integrated into the workflow and work practices of GPs and practice nurses. Before each consultation in the clinic, the patients filled out a written consent form to allow the first author to participate in the consultation. Approximately 50 h of fieldwork were conducted, and jottings were taken during observations [21].

### Semi-structured interviews

Five of the GPs in the above clinics (two women and three men, between 35 and 70 years of age) were interviewed during working hours (audio-recorded interviews with a duration from 20 to 30 min), using a semi-structured interview guide containing open-ended questions relating to their experiences with email consultations (general experiences but also with the email consultations of the day) and reflections on the relational potential of email consultations. 11 additional semi-structured interviews were conducted with GPs (five women and six men, between 43 and 59 years of age). In selecting GPs for these interviews, variations were strived for regarding the GPs’ age, gender, practice type, geographical location and years of practice as a GP (see Table 1 for full data overview). Participants were from a broad geographic distribution including participants from four of Denmark’s five geographically defined regions. All 11 GPs were given the option to choose between a face-to-face or a telephone interview. Seven GPs chose the telephone option. All interviews were audio-recorded and lasted from 45 to 60 min. The recruitment of GPs continued until no further information or interpretation of the relational potential of email consultation were found, commonly referred to as saturation [22].

**Table 1.** Data overview.

Participants	Gender	Age	Practice type: group or solo	Practice location and number	Type of participation
1	Male	70	Group	Rural	Observation + interview
				Practice #1	
2	Male	39	Group	Rural	Observation + interview
				Practice #1	
3	Male	35	Group	Rural	Observation + interview
				Practice #1	
4	Female	56	Group	Rural	Observation + interview
				Practice #2	
5	Female	50	Solo	Urban	Observation + interview
				Practice #3	
6	Male	69	Group	Urban	Observation
				Practice #4	
7	Female	59	Group	Urban	Interview (Tel.)
8	Female	47	Group	Urban	Interview (Tel.)
9	Female	42	Group	Urban	Interview
10	Female	50	Group	Urban	Interview
11	Male	45	Group	Urban	Interview (Tel.)
12	Male	47	Group	Rural	Interview
13	Male	42	Group	Rural	Interview
14	Male	49	Group	Urban	Interview (Tel.)
15	Male	48	Group	Rural	Interview (Tel.)
16	Male	43	Group	Urban	Interview (Tel.)
17	Female	53	Group	Urban	Interview (Tel.)

### Data analysis

Jottings from the fieldwork were worked up into detailed descriptive, first-person field notes including informal conversations. All interviews were transcribed verbatim and both interview transcripts and reports with fieldwork notes were analyzed by means of the software program NVivo 12 Pro. The first author coded the transcripts in two phases: an initial open coding and a subsequent closed thematic coding focusing on identified themes and subthemes allowing for expansion and reduction along the way (see Table 2) [23]. By means of the inductive thematic analysis, we aimed to stay as close as possible to the meanings in the data although we are aware that analysis is always shaped by the researchers’ assumptions, professional training, personal and political standpoints, etc. All authors discussed and agreed upon the identified themes (intercoder agreement), relating them to the original transcripts and aligning them where necessary. The authors acknowledge that their position within communication, media and medical sociology might have biased them towards a positive view on human interaction through technology. However, a part-time GP, drawing from experiences from many years of clinical work in a general practice clinic, was part of the author team and theoretical assumptions and ideas were challenged and discussed critically throughout the analytic process.

**Table 2.** Overview of nodes\focused coding.

Code names	Description
GP-perspectives on relational potential	Overarching theme
Priviledging f-t-f and telephone con.	Main theme
Interpretation difficulties The doctor’s insecurity Poorer communication quality Redirecting to other consultations (f-t-f or telephone)	Subthemes
Mutual knowledge as prerequisite	Main theme
Confidence regarding econ administration Mutual trust and respect Interpersonal continuity and continuity of care Difficulties with colleagues’ emails (decontextualized emails)	Subthemes
GP-experiences with relational potential	Overarching theme
Supportive communication channel	Main theme
Psycho-emotional support Inducing hope Psychiatric patients Keeping the relationship warm	Subthemes
Relationship-oriented email	Main theme
Non-medical issues Expressing gratitude GPs showing compassion and empathy GPs showing interest	Subthemes
Email as an outlet for affective expression	Main theme
Outlet Anxiety disorders Therapeutic writing Voicing dissatisfaction and anger	Subthemes

### Ethical considerations

Upon giving written informed consent, all participants were informed that participation in the study was voluntary and that data were kept and secured in accordance with the General Data Protection Regulation (GDPR) and the Declaration of Helsinki [24]. The study was approved by the institutional review board of the University of Southern Denmark: *The Research and Innovation Organization* (RIO) (Journal no. 10457) and permission to store and analyse the data was granted.

## Results

Five themes were identified: (a) Privileging face-to-face and telephone consultations, (b) Mutual knowledge as a prerequisite, (c) Supportive communication channel, (d) Relationship-oriented email consultation and (e) Email as an outlet for affective expression.

### Privileging face-to-face and telephone consultations

From the way that most of the GPs managed and organised email consultations in the clinic or talked about them in the interview setting, it was evident that email consultation was not conceptualized as a medium for unfolding socio-emotional exchanges in the context of the doctor–patient relationship. All GPs stated that for matters that went beyond routine medical tasks, they much preferred face-to-face consultations, highlighting the value of patient–physician interaction and human contact in person. One male GP described it thus:

Of course I much prefer to have the patients right in front of me, because this is how I prefer to be a doctor because then I am able to look my patients in the eyes - and to see how they look and how they move, their mimics and so on…and this is a big part of the communication. I miss some of that in a telephone consultation and in an email consultation, I don’t get that at all. The email consultation is very impersonal and difficult to interpret, (Male GP 10).

According to the GP in the above excerpt, email consultation does not provide the subtle information about the patient’s health and well-being that can normally be observed from the patient’s physical appearance and that aids interpretation. Thus, the GPs’ predilection for using face-to-face consultations as a platform for interactional practices is closely tied to the perceived interpretation possibilities that the human face-to-face encounter is experienced to provide, and to concerns about missing out on this valuable information when using email consultation. For that reason, the GPs participating in this study displayed a consistent tendency to wanting to redirect email consultations with emotional and complex content to either face-to-face or telephone consultations. This overall tendency also became apparent during the fieldwork, for example in the way one of the male GPs from one of the larger clinics chose to manage an email consultation from one of his patients:

In between the consultations and using a shortcut on the computer, the GP enters the inbox of the clinic with a single maneuver and with a quick glance on the messages received, he opens one of them, telling me that this is a message from one of his “functional patients” who he has seen many times and who is now writing to him in order to get a re-referral to her psychiatrist. The GP explains that normally he is not allowed to refer patients on the basis of an email consultation, but in this case, he would actually be okay with making the referral, because he knows the patient well and knows what she is struggling with. However, instead of just writing back to her about the referral, he chooses to call her: “I prefer to give her a call to check up on her and hear how she’s doing – just in case.” (Fieldnotes, day 3, clinic 1).

The excerpt supports the overall finding that when dealing with complex, emotional and sensitive matters other consultation forms than email consultation are preferred by the GPs. In the above excerpt, the GP provides some context as to why he chooses the telephone medium to respond to the patient’s email consultation: the telephone will provide him with more information about the patient’s well-being and mental state, enabling him to listen to vocal cues and affective tone. The above excerpt can also be used to illustrate how email consultation is one element of an ‘interaction package’ [9] that consists of different opportunities to communicate and, furthermore, that the GP is confronted on a daily basis with situations in which choices are made about which communication medium is the most appropriate to use in particular situations with particular patients.

### Mutual knowledge as a prerequisite

Some GPs signaled more openness towards unfolding complex matters through email consultation than others, stating, however, that they were selective in choosing which patients they would communicate with about socio-emotional and sensitive issues. Knowing the patient well was for most of the GPs perceived as a prerequisite for unfolding this kind of communication through an email consultation. A male GP explains:

I’d say that it requires that you have a prior knowledge of the patient. There are some of my patients who I know well and who I am confident are able to administer it. The relationship is important, I mean, that there’s mutual trust and respect. So, there has to be some kind of initial dynamic in place first, I think. (Male GP 5).

The overall point emphasized in the above excerpt is that communicating with patients through email consultation about matters that go beyond the typical information-delivery messages requires a thorough knowledge of one another, for example, familiarity with the patient’s emotional needs and clinical situation, as well as feelings of mutual trust and respect. Relating to this point, the importance of the patient knowing the doctor, including his/her writing style, was stressed by one of the GPs: ‘knowing as well how they will read *my* words – that is, after all, also extremely important in all this’.

During fieldwork, it also became apparent how the GPs’ acceptability of email consultation was to a large extent dependent on experiences of interpersonal continuity and continuity of care. In one of the larger clinics, the email consultations were organised in such a way that they were received in a shared inbox that the whole clinic team could access. The perceived challenge of responding to email consultation from patients that ‘belonged’ to one’s colleague was exemplified in a conversation around the lunch table:

At lunch (five GPs, two nurses and a secretary are present), one of the GPs vents her frustration over how difficult she thinks it is to respond to email consultations from her colleague’s patients. She explains that just before going to lunch (she arrives 20 minutes into the break), she has tried to label today’s email consultations from the shared inbox with the initials of the doctor that the patients are mostly choosing as their personal doctor. As one of the GPs is currently on holiday, she has tried to answer the email consultation pertaining to him however with difficulty and insecurity. The colleagues recognize her frustration, adding to this, that it is very time-consuming to figure out what the patient is writing about when they have not been prepared from a prior physical meeting for what the patient is writing about, let alone if they do not know the patient. They continue talking about the policy of the clinic: that every patient should be encouraged to choose his/her personal doctor and that email consultation, where possible, ought to be directed to that particular doctor, (fieldnotes, day 1, clinic 3).

The above excerpt can be seen as illustrative of daily practice scenarios where the management of email consultation is challenged as a result of an email consultation use that decontextualizes it from the relationship. Seeking to find a model whereby each email consultation is forwarded to the ‘personal’ doctor can be seen as an attempt to recontextualize email consultation within the doctor–patient relationship, hereby signaling a view of email consultation as something that requires a prior relational dynamic and foundation.

### Supportive communication channel

By those GPs who did use email consultation for other purposes than task-oriented communication with patients, they knew well, email consultation was experienced to serve as a supportive communication channel. Patients perceived to particularly benefit from this supportive email communication were patients who were challenged or distressed, either on a regular basis because of permanent life circumstances (cognitive impairment, anxiety disorders, drug or alcohol abuse) or more episodically when confronted with loss (e.g. death in the family) or other challenges in life (divorce, stress, job loss, surgery, etc.). Responding to patients’ email consultations in a reassuring, comforting and understanding manner was thought to contribute to relieving some of the sufferings of the patient. For example, one GP talked about how email consultation could be used to provide ‘vicarious hope’ responses in which the GP conveys an understanding of the patient’s distress and vulnerability, express a hopeful attitude towards the patient and signal partnership:

The email consultation can be used for providing what we call “vicarious hope”, where the patient is being met in his/her frustration and feeling of powerlessness and where I can make a therapeutic intervention through email, writing that I think that the patient should book an appointment but that I am convinced that everything is going to be alright and that we will make a plan together for how to move on. And then the email consultation becomes a dialogue over email, where we write a little together until the patient comes to me. (Male GP 6).

As can be seen in the above excerpt, using email consultation as a supportive communication channel allows the GP to meet the patients’ more immediate socio-emotional and psychological needs until the face-to-face appointment. As such, communication through email supplements rather than substitutes face-to-face consultations.

### Relationship-oriented email consultation

A few GPs described some of their email consultations as concerning ‘only’ the doctor–patient relationship, meaning that these email consultations were without a direct link to a clinical or medical issue requiring medical action (e.g. information-delivery) but more of a relationship-oriented kind. Patients writing these messages would be providing the GP with updated information about their well-being or health status, for example, following a specific treatment, sick leave or some other challenging event or period in their life or simply telling the GP how their weekend went. In doctor–patient relationships where this type of exchange went well, the GP would typically deliver responses of the kind: ‘I’m really glad to hear that you are now feeling better’, or ‘I’m happy to hear that you spent a nice weekend’. The GPs receiving these kinds of emails from their patients were accepting of them as long as they satisfied their patients’ needs, acknowledging at the same time that strictly speaking, email consultation was not intended for this relationship-oriented communication. However, as one of the GPs reasoned, one could nonetheless argue that this type of email consultation was part of the core functions of general practice:

And you could say, if we are to rationalize practice, then these email consultations should go out, but…we also talk about general practice as a place where we should comfort, relieve suffering and heal, and in the mentioned order. (Male GP 6).

A relationship-oriented email consultation was also expressed in those messages in which the patient would express his/her gratitude and thanks for the care received – a message that, according to the GPs, was easier for the patients to express through an email than in the direct encounter or through the telephone. On the side of the GPs some of them were also initiating messages to the patients with the purpose of showing compassion and caring for their patients who underwent difficult life circumstances:

Sometimes, I write out of interest: “How did it go with the examination that you were so nervous about – did you do alright?” or: “I’m sorry to hear that you broke your hip,” or whatever… So, I also write to them sometimes…and they are happy about that, (female GP 4).

Experiencing that the patients were generally happy to receive email consultations initiated by their GP that expressed a kind of affective presence towards their life seemed to be a source of joy to the GP him/herself and to ‘oil the gears’ of the doctor–patient relationship. As we shall see in the following theme, email consultation was also described to be used as a medium for other kinds of affective expression.

### Email as an outlet for affective expression

Some GPs talked about their experiences with patients using email consultation as an outlet for expressing an internal emotional or affective state. Examples were given of patients suffering from anxiety disorders who, for example, in the middle of the night, used email consultation as a way to express their sufferings and frustrations. Some of the GPs noted that expressing emotions and affect in this way through writing served a therapeutic function: as an immediate way to express oneself to someone who reads and responds with empathy help people better understand and subsequently regulate their affect:

I have experienced several mails of the type where everything shuts down in the middle of the night and where people have it formulated and sent down to me and then they know that I read it the next morning and write to them and in some way or another, it’s like an outlet for them, in the situation that they are in. (Male GP 11).

Email consultation was also used as an outlet for expressing negative affect and emotions towards the GP, for example, anger or dissatisfaction with the treatment provided. Some of the GPs noted that communicating remotely about matters of disagreement was easier for patients than up-front confrontations and facilitated communication that was more thought out than face-to-face communication would sometimes be. However, examples of harsh emails were also given in which GPs were being scolded by their patients who were venting their frustrations and anger when writing. One GP gave an example of an email received just recently from a patient conveying the information that the patient had just filed a complaint:

Then a patient wrote to me yesterday: “Now I have filed a complaint against you because the process around my back has been so dragged out and now, I got fired from work. I just wanted to tell you that. Regards B.” (Female GP 2).

These types of emails were experienced as distressing, making the GPs use a lot of time weighing their words when responding to the patients and needing support from colleagues regarding how to control ones’ own affect when responding and handle them in a professional way.

## Discussion

### Principal findings

This study explored and analyzed Danish GPs’ perspectives on and experiences with communication through email consultation that serve specific relational purposes. The principal findings were that GPs are mainly attributing the relational potentials and functions to face-to-face or telephone consultations because of the way that they provide opportunities to observe patients’ emotionally charged body language or non-verbal cues, aiding the clinical interpretation of symptoms, signs and their meanings. The point that is central to this perspective is that the GPs, in privileging face-to-face- and telephone consultations, are motivated by concern over the quality of care that the email medium enables them to provide. Overall, the findings exemplify a general feature present in much of the collected material: that GPs are highly sensitive to the opportunities for interpretation that the different consultation forms provide, in many cases judging email consultations unsuitable for complex communication processes. This might reflect a reality in which a gap exists between patients and their GPs: patients increasingly use secure web portals to communicate with their providers but GPs have not been trained in digital communication (though being well trained in face-to-face communication and the consultation process) and tend to focus more on weaknesses than opportunities of this kind of communication [25].

### Relation to other studies

The GPs’ predilection for using email consultation for routine or technical tasks supports existing literature documenting that email consultation is primarily used as an information-delivery tool, not a medium for relationship deepening or maintenance [9]. Interestingly, however, redirecting patients to the GPs’ preferred and ‘safest’ mode of interaction might not necessarily satisfy their patients. Research documents that some patients have been found to be more comfortable bringing up sensitive or difficult issues online than in face-to-face consultations [26] and that the asynchronicity of place afforded by email may free patients from the social constraints of the patient role [4]. Furthermore, the burgeoning field of ‘existential media studies’ [27] points to the key role played by social media in sharing affect and emotions online. In connection with illness experiences, a growing number of patients use the internet for interactive services: engaging in peer communities, sharing personal stories and providing peer support [28]. Against the backdrop of an increasingly digitally engaged patient population, also at an experiential level, there is reason to believe that patients also wish relational exchanges within the physician–patient relationship to take place online, especially with the GP that they know well as a result of the list system that prompts relational continuity.

As the findings of this study show, many patients wish to satisfy not only their biomedical but also their psychological and social-emotional needs through email consultation, hereby challenging how the GPs of the present study ‘prefer to be a doctor’. The GPs’ a priori reluctance to engage in relational exchanges with patients through email consultations can be further understood with reference to computer-mediated communication (CMC) theories. Social presence theory and the ‘cues-filtered-out’ theories influencing CMC research in the 1970s and 1980s both rely on the assumption that the fewer codes and channels available within a medium, the less attention is paid by the user to the social presence of participants [29,30]. As CMC has a reduced capacity to transmit information about participants (social and context cues), social presence is said to be extremely low in comparison to face-to-face communication which will result in predictable negative effects on relational aspects of communication. Important implications of this view are that CMC should focus solely on instrumental tasks rather than on social interaction (which in itself steers towards impersonal, rather than interpersonal, communication) and that such relational effects are inherent, constant and context-invariant [31]. Although the perspectives of the GPs of this study resonate with the afore-mentioned ideas, for example, that face-to-face communication will always be of higher relational and socio-emotional value as a result of higher social cues transmission, the practice accounts of the GPs of this study also represent a broader reality by showing a context-variant use of the technology. The GPs show awareness of how email consultation might have positive effects on the doctor–patient relationship pointing beyond the digital contact when used in conjunction with office visits and with the ‘right’ people.

An interesting aspect of the findings involves how the GPs select patients for email communication depending on the length of the relationship (longitudinal care), the quality of the doctor–patient relationship (the depth of the relationship), and whether or not the GPs are confident about the patient’s ability to ‘administer it’. These findings are in line with existing research and fit into the core narratives of general practice about the value of relational continuity and continuity of care within the context of the doctor–patient relationship [32].

Another relevant finding of this study relates to how some of the GPs used email consultation as a medium for relationship-centered caring and for demonstrating their ability to be ‘humane’ professionals. These voluntary, proactive relationship-fostering actions can be understood as a need of the GP to maintain and support the personal doctor–patient relationship in a healthcare system where constraints of the organisational context might decrease the time for relationship-building and increase the risk of transforming doctoring to assembly-line medicine [33]. As argued by Baur [9], when used in conjunction with traditional office visits (having a mean time of ten minutes), email consultation may constitute an important supplementary element in doctor–patient relationships in which the GP wishes to provide both technical, bio-medical expertise and human care.

### Strengths and weaknesses

Because subjective perspectives and experiences of GPs in connection with daily uses of email consultation have been largely unstudied, the qualitative nature of this study has particular strengths. Whether patients perceive that email consultation provides them with emotional support is an unaddressed matter in this study. Reporting only the GPs’ perspectives and experiences is thus a limitation. A further strength of this study is the multidisciplinary author group representing the humanities (media studies and medical sociology) as well as the medical sciences, hereby eliciting rich and manifold views and discussions.

### Meaning of the study

In a time when more doctor–patient interaction becomes digital, it is relevant for GPs to learn more about how email consultations might also be used to develop, maintain and/or dissolve the relational axis of the doctor–patient relationship. Future research needs to concentrate on whether and how email consultation, and other computer-mediated doctor–patient communication, for example, video consultations, can be used effectively and meaningfully, not only for simple information-delivery but also for more complex relational purposes. This includes a focus on the development of more systematic training and education for GPs and other health care professionals in digital communication, including learning strategies to reach a mutual understanding between GP and patient about how to use email consultations in each doctor–patient relationship.

## Conclusion

Knowledge of the patient seems to be a vital factor for the GP’s comfort in and acceptability of relational functions of email consultation. In relationships of mutual respect and trust, email consultation can be used to provide feedback to health concerns and to offer hope and reassurance. In these cases, saving time is not the dominant rationale but satisfying the patient’s emotional needs and fulfilling the need of the GP him/herself of acting as the personal, empathic GP. In a time where the personal doctor–patient relationship is challenged due to organisational restraints, more attention should be paid to the relational potential of email consultation, and of computer-mediated communication overall.
